# Hemocyte-hemocyte adhesion by granulocytes is associated with cellular immunity in the cricket, *Gryllus bimaculatus*

**DOI:** 10.1038/s41598-019-54484-5

**Published:** 2019-12-02

**Authors:** Youngwoo Cho, Saeyoull Cho

**Affiliations:** Department of Applied Biology, College of Agriculture and Life Science, Environment Friendly Agriculture Center, Kangwon National University, Chuncheon, Republic of Korea

**Keywords:** Phagocytes, Entomology

## Abstract

In this study, more than 1,000 cricket (*Gryllus bimaculatus*) hemocytes were classified based on their size and morphology. These hemocytes were classified into six types: granulocytes, plasmatocytes, prohemocytes, spherulocytes, coagulocytes, and oenocytoids. Hemocyte cultures was observed in real time to determine which hemocytes were associated with cellular immune responses against potential pathogens. Granulocytes were identified as the professional immune cell that mediates nodulation, encapsulation, and phagocytosis of pathogens. Granulocytes have been shown to actively produce various sticky nets (amoeba-like hairs and extracellular traps) from their plasma membranes that they use to gather other hemocytes and to implement cellular immune responses. The activation of lysosomes in granulocytes started at 4 h, peaked at 12 h, and returned to baseline by 24 h post-infection. At 48 h post-infection, cells could be found within the cytoplasm of granulocytes and reactivated lysosomes surrounding these cells were visible. This result seems to reflect a phenomenon in which necrotic granulocytes are removed by other healthy granulocytes. This unique mechanism of cellular immunity is therefore a way to efficiently and effectively remove pathogens and simultaneously maintain healthy hemocytes.

## Introduction

The immune response in insects is classified into the cellular immune response, which is mediated by insect blood cells (hemocytes), and the humoral immune response, which is mediated by various effector molecules, including antimicrobial peptides (AMPs) and the phenoloxidase (PO) cascade^1–5^. To date, seven types of hemocytes have been identified in various insects: prohemocytes, plasmatocytes, granulocytes, spherulocytes, adipohemocytes, coagulocytes, and oenocytoids^6,7^. However, most insects do not have all seven types. For example, in the case of the fly and the mosquito, only three types of hemocytes have been described, while in many species of Coleoptera there are at least five types of hemocytes^2,8–10^. Furthermore, even in insects with many types of hemocytes, only a few types are thought to be key players in cellular immunity. Plasmatocytes and granulocytes, for example, are involved in cellular immunity through their phagocytosis of potentially hazardous organisms *in vivo*, which is similar to the function carried out by monocytes, neutrophils, and eosinophils in humans. Moreover, insect immune cells directly produce or secrete antibiotic/antimicrobial proteins to kill invading microorganisms, which is also a function carried out by neutrophils in humans. The silk moth (*Hyalophora cecropia*) was the first insect reported to produce antibiotic proteins^11^. It has recently been reported that antibiotic proteins are secreted by the hemocytes of the blue blowfly (*Calliphora vicina*)^12^. However, insect plasmatocytes and granulocytes do not perform the functions of human dendritic cells, B cells, or T cells with respect to pathogen tagging or antibody production^13,14^.

Immunological activation of hemocytes in insects begins with a morphological change in the plasma membrane. For example, in the case of white-spotted flowers, the hemocytes begin to enlarge within 12 h of bacterial infection, and the cell membrane begins to form fan-like or amoeba-like structures^8^. When insects are infected with parasites or other microorganisms, the immune cell membrane forms irregular nets that surround the invading pathogens and remove them through encapsulation, nodulation, and phagocytosis^15–18^. Similar changes in the cell membrane are commonly seen in infected mammalian neutrophils and are referred to as neutrophil extracellular traps (NETs). NETs grow to approximately 50 μm in length, and are composed of not only diverse proteins, but also neutrophil DNA^19^. NETs are important structures that help neutrophils to efficiently capture and kill pathogens^20^. The formation of nets and the release of DNA by oenocytoids has been reported in insects^21^.

After activation, aging immune cells eventually lose their function and are removed from the circulation. Aging immune cells can be removed by autophagocytosis, apoptosis, or necrosis, depending on the type of involved pathogen and host immune system^10,22–27^. Neutrophils die after NETs formation through a mechanism called NETosis because it occurs specifically after NETs formation. Unlike necrosis and apoptosis, NETosis is characterized by the formation of vacuoles and occurs without extracellular exposure of the phosphatidylserine in the cell membrane^28^. There have been no reports of NETosis in insects.

Insects live in diverse environments. Crickets in particular live in moist and mild environments where they frequently encounter pathogenic microorganisms. We therefore hypothesized that cellular immunity is likely to be well-developed in the cricket. This study aimed to characterize cellular immunity in the cricket, *Gryllus bimaculatus*. We identified the major types of immune cells in crickets and investigated their main functions and characteristics. Net formation and cellular immune responses such as phagocytosis, nodulation, and encapsulation by plasma membrane nets were observed. In addition, immune cell death was observed after activation by pathogens.

## Results

### Types of hemocytes in crickets

**Figure 1:**
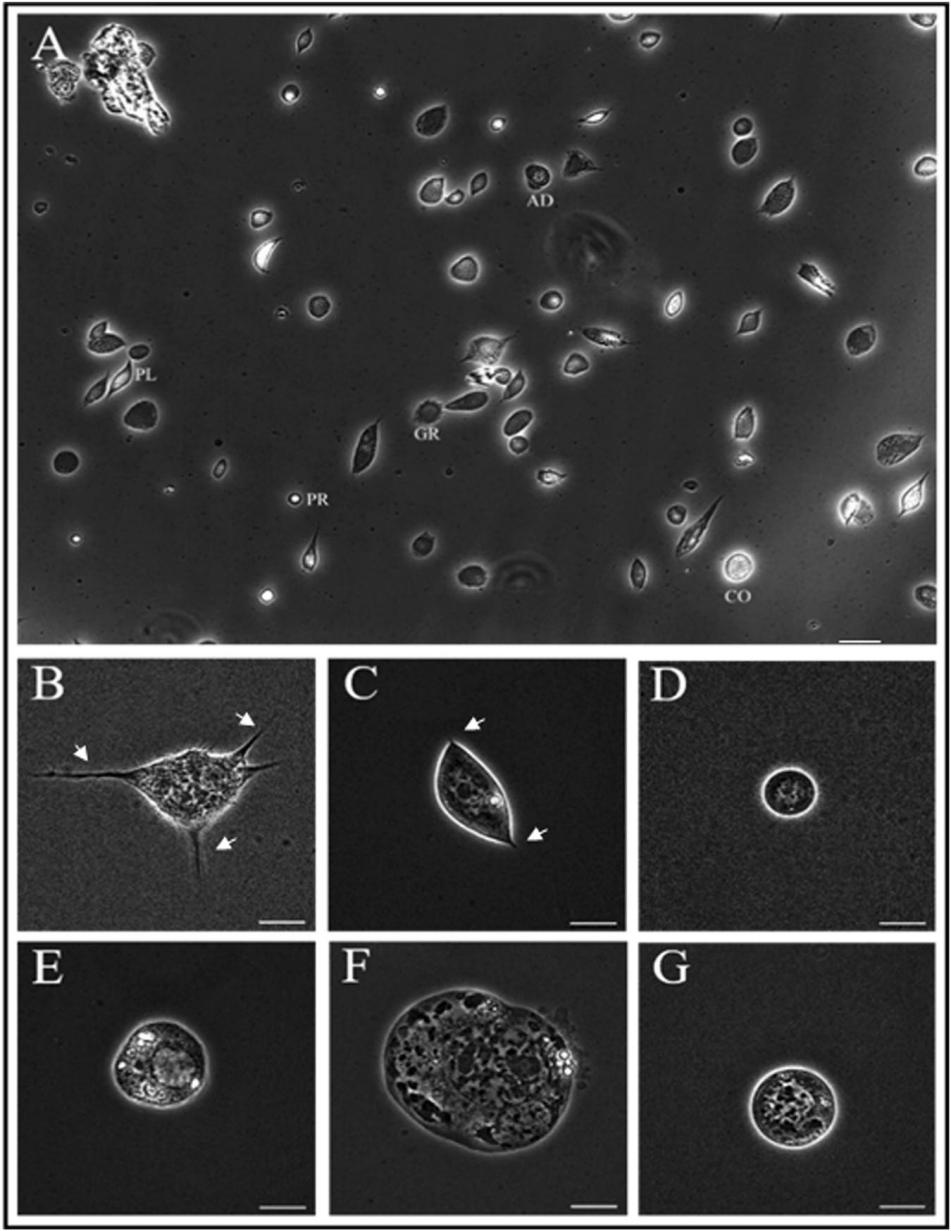
The six types of hemocytes observed in the blood of crickets. Overall shape and relative size of hemocytes in the hemolymph (**A**). The six types of hemocytes found in *G*. *bimaculatus* were classified as granulocytes (**B ; GR**), plasmatocytes (**C ; PL**), prohemocytes (**D ; PR**), coagulocytes (**E** ; CO), oenocytoids (**F ; OE**), and spherulocytes (**G ; AD**) based on size and morphology. The fan-like or amoeba-like structures on the plasma membrane of granulocytes and plasmatocytes are indicated by white arrows (**B** and **C**). Scale bar = 25 µm (**A**) or 10 µm (**B**~**G**).

Figure 1A shows a differential interference contrast (DIC) microscopy image of cricket hemocytes, showing cells of various sizes and shapes. For accurate identification of these hemocytes, approximately 1,000 cells were classified by their size and morphology (data not shown) into six types (Fig. 1B~G). As shown in Fig. 1B, the typical granulocyte was observed in the hemolymph. The granulocytes were usually round or oval in shape, and many polymorphic granules were observed within the cytoplasm. Small pseudopodia or filopodia were observed on the granulocyte plasma membrane (Fig. 1B, indicated by white arrows). The cell shown in Fig. 1C was considered to be plasmatocytes due to their typical spindle shape and the long, hair-like structures observed at both ends of the plasma membrane (indicated by white arrows). Figure 1D shows a prohemocyte, which are the smallest round cells observed in the insect hemolymph. The nuclei were large relative to the cell size and were located in the middle of the cell (Fig. 1D). Thus, the cytoplasm and the nucleus of the prohemocytes were often indistinguishable. Figure 1E depicts a coagulocyte containing various cytoplasmic granules. The plasma membranes of the coagulocytes were smooth, without any structures, and their nuclei were large and easily observed. The oenocytoids were the largest type of hemocyte observed and were scarce in the hemolymph (Fig. 1F). The oenocytoids usually had a round fried egg shape with numerous cytoplasmic granules. Figure 1G shows a spherulocyte, which were round and medium in size with many small dark or shining granules in the cytoplasm. As shown in Fig. 1D~G, no structures were visible on the plasma membranes of the prohemocytes, coagulocytes, oenocytoids, or spherulocytes, which were optically very smooth.

### Nodulation and encapsulation by hemocytes

In insects, cellular immune responses such as nodulation, encapsulation, and phagocytosis are performed by immune hemocytes such as granulocytes and plasmatocytes. After pathogen exposure, these hemocytes change their shape and develop large amoeba-like or fan-like structures in their plasma membranes. To observe these rapid morphological changes in real time, we developed a technique that allows us to culture insect hemocytes for 7 days while preserving physiological activity (described in the Materials and Methods). Although we could not establish stable hemocyte lines that can be passaged for several years, we were able to observe the morphology of hemocytes in response to pathogens *in vitro*. Supplementary Movie C shows only hemocytes after 12 h of incubation. Most cultured cells were actively moving. Some cells exhibited nets around the cell membrane during culture and were found to be attached to the culture slides. The hemocytes rarely aggregated or formed large clusters.

**Figure 2:**
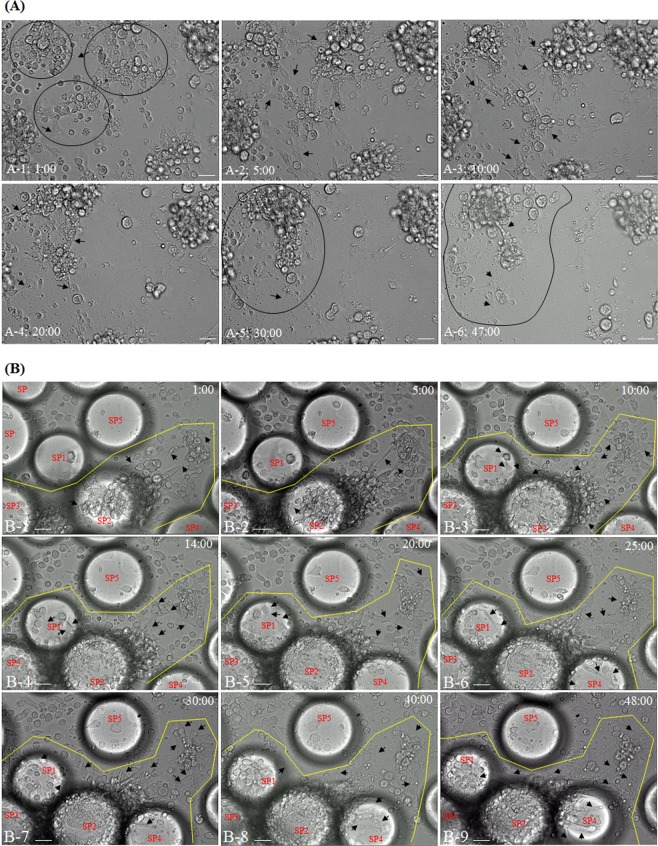
Live-cell images of cricket hemocytes infected with *E*. *coli* or Sephadex beads. (**A**) Light microscope images showing hemocytes cultured with *E*. *coli*. As the incubation time increased, nets (amoeba-like hairs or extracellular traps; indicated by black arrows) were produced by specific hemocytes. All six types of hemocytes were aggregated into large clusters by these nets (A-1~A-6; indicated by black boxes). Movie available as Movie 1 (time in minutes post-inoculation). (**B**) Light microscope images showing hemocytes cultured with Sephadex beads. Sephadex beads around the hemocytes were randomly labeled SP1, SP2, SP3, SP4, and SP5. Over time, the Sephadex beads (SP1, SP2, SP3, and SP4) became surrounded by hemocytes and encapsulated by various types of nets (B-1~B-9; indicated by black arrows). Movie available as Movie 2 (time in minutes post-inoculation). Scale bar = 25 µm (**A** and **B**). Movie C shows hemocytes cultured alone, without Sephadex beads. Most hemocytes were actively moving. A few cells were attached to the culture slides by plasma membrane nets. However, large clusters of hemocytes were not observed.

**Figure 3:**
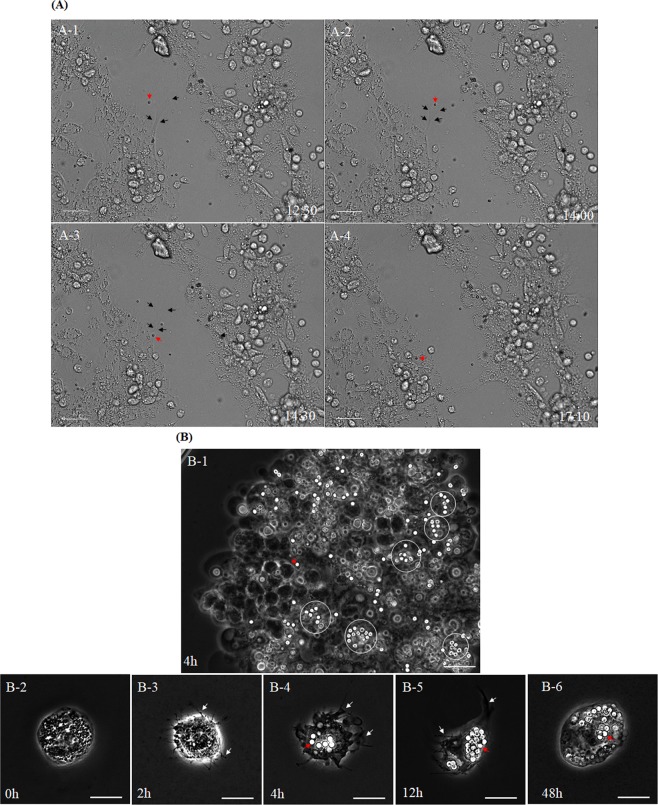
Live-cell images of granulocytes stimulated with carboxylate-modified polystyrene latex beads. (**A**) Light microscope images showing hemocytes cultured with carboxylated-modified polystyrene latex beads for 12 h. A-1~A-4, cultured hemocytes after 12~18 min. The nets produced by the hemocytes (black arrows) could be seen forming around the latex beads (indicated by red arrows). The latex beads were engulfed by the nets and eventually were captured within the cytoplasm of the hemocytes (A-4). Movie available as Movie 3 (time in minutes post-stimulation). Scale bar = 40 µm. (**B**) Magnified images. (B-1) After 4 h, many carboxylate-modified polystyrene latex beads were captured by hemocytes (latex beads, red arrows; and specific hemocytes, white circles). (B-2~B-6) Cultured granulocytes at 0, 2, 4, 12, and 48 h post-stimulation. (B-2) Resting granulocytes, which are round or oval in shape. (B-3) At 2 h post-stimulation, the granulocytes began to show morphological changes, and fan-like or amoeba-like plasma membrane structures (white arrows) can be seen. (B-4 and -5) A large number of latex beads had accumulated in the cytoplasm of granulocytes at 4 and 12 h post-infection. (B-6) By 48 h post-infection, many latex beads had accumulated in the granulocyte cytoplasm, but nets were no longer observed, and many granulocytes seemed to be inert. Scale bar = 15 µm (B-1) or 10 µm (B-2~B-6).

**Figure 4:**
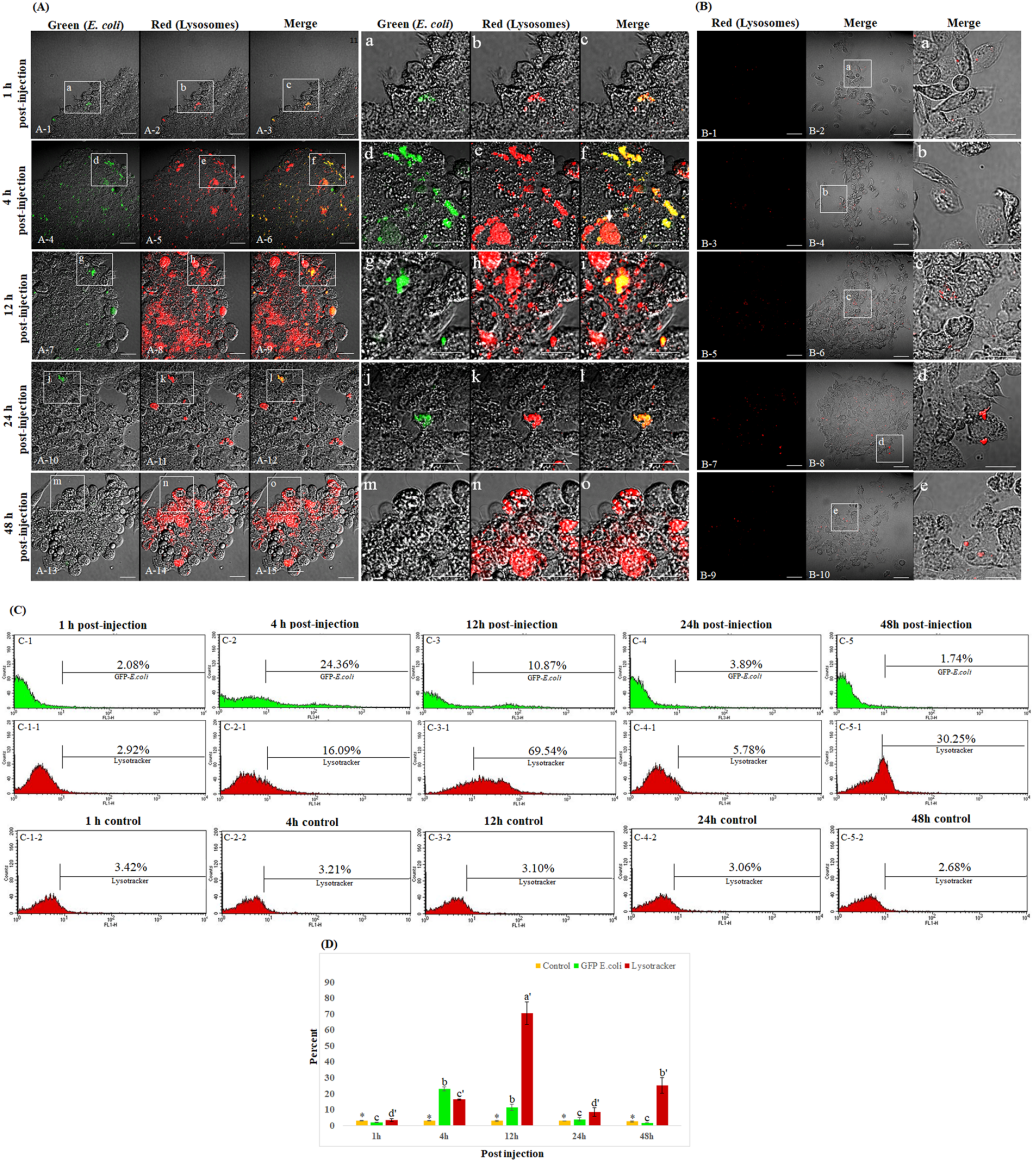
LysoTracker Red labeling of granulocyte lysosomes in crickets injected with green fluorescent *E*. *coli* particles. (**A**) Development of granulocyte lysosomes at 0 h, 4 h, 12 h, 24 h, and 48 h post-injection of *E*. *coli* particles. (A-1, A-4, A-7, A-10, and A-13) The *E*. *coli* particles, which are used as markers of phagocytosis, fluoresce green when they reach acidified organelles such as intracellular lysosomes. (A-2, A-5, A-8, A-11, and A-14) Confocal fluorescent microscope images of granulocytes stained with LysoTracker Red (a lysosomal marker). (A-1 and A-2) The green and red fluorescent signals could be observed in the granulocyte cytoplasm beginning at 1 h post-injection. (A-4 and A-5) Many granulocytes showed green and red fluorescence in the highly polymorphic vacuoles of granulocytes at 4 h post-injection. (A-7 and -8) At 12 h post-injection, the green fluorescent signal had dimmed but the red fluorescent signal remained. (A-10 and A-11) At 24 h post-injection, the green and red fluorescent signals had both almost disappeared. (A-13 and A-14) At 48 h post-injection, the green fluorescent signal had completely disappeared but the red fluorescent signal was observed again. Merged images of the green and red fluorescent signals are shown (A-3, A-6, A-9, A-12, and A-15). (a~o) The insets in panels A-1 ~A-15 (indicated by white boxes) at higher magnification. (**B**) The red fluorescent signal in granulocytes from crickets that were injected with PBS (negative control). (**C**) Flow cytometric analysis at 1 h~48 h post-injection. (C-1 and C-2) The green fluorescent signal was 2.08% at 1 h post-injection and increased to 24.6% at 4 h post-injection. (C-3~C-5) The green fluorescent signal gradually decreased, to 10.87% at 12 h, 3.98% at 24 h, and 1.74% at 48 h. (C-1-1~C-4-1) The red fluorescent signal increased to 69.54% at 12 h post-injection and decreased to 5.78% at 24 h post-infection. (C-5-1) The red fluorescent signal increased again, to 30.25%, at 48 h post-injection. (C-1-2~C-5-2) The red fluorescent signal in granulocytes from crickets that were injected with PBS buffer only. (**D**) The flow cytometry analyses were repeated three times.

**Figure 5:**
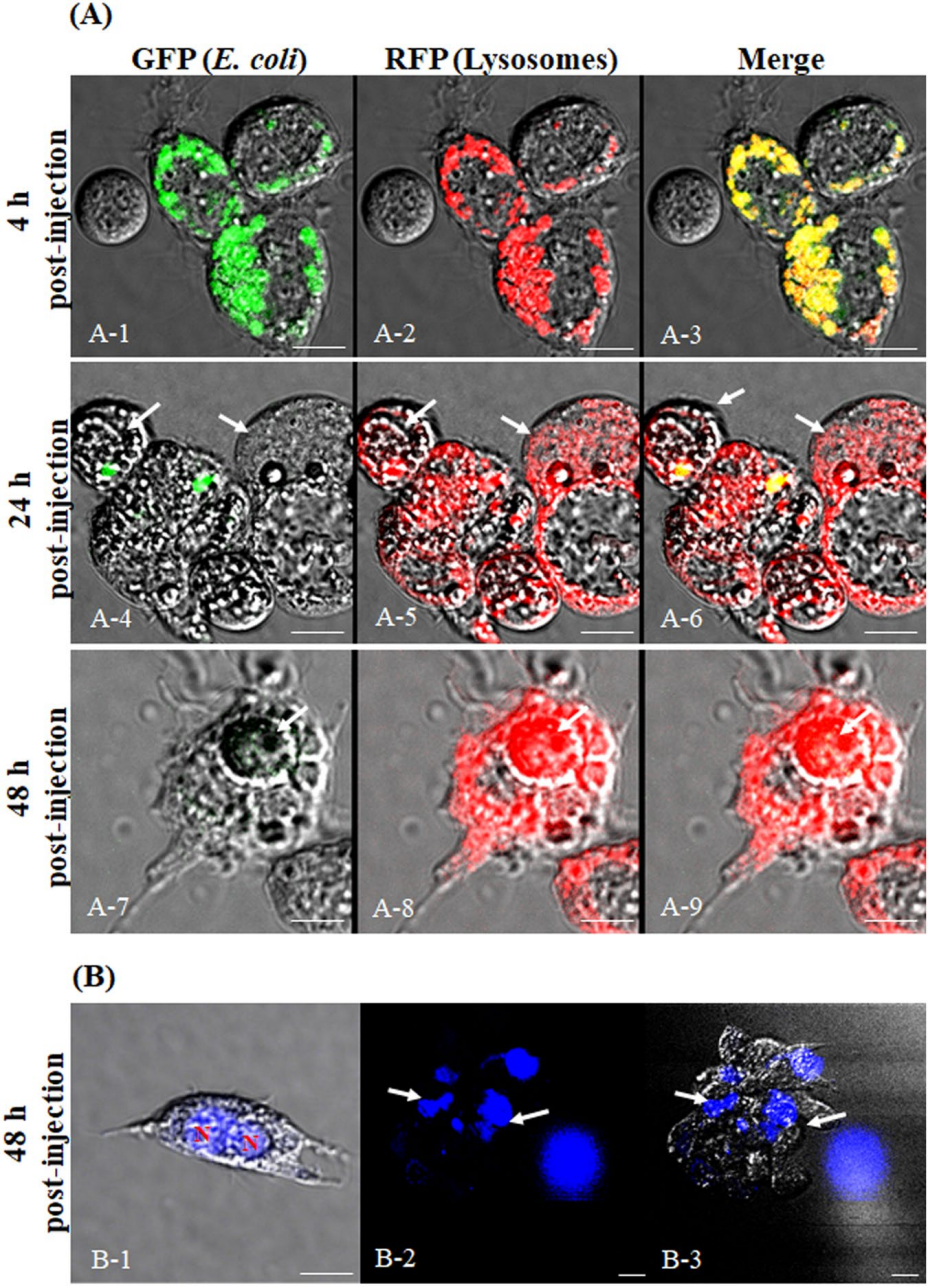
Reactivation of granulocyte lysosomes at 48 h post-injection. (**A**) Fluorescent microscope images of granulocytes at 4 h, 24 h, and 48 h post-injection. (A-1 and A-2) Granulocytes showed green and red fluorescent signals in the highly polymorphic vacuoles at 4 h post-injection. (A-4 and A-5) At 12 h post-injection, the green fluorescent signal had dimmed, but the red fluorescent signal remained. In addition, some cells were observed within the cytoplasm of the granulocytes (white arrows). (A-7 and A-8) This phenomenon was more frequently observed at 48 h post-injection. In addition, the lysosomes surrounding these engulfed cells were activated (A-8). Merged images of the green and red fluorescent signals are shown (A-3, A-6, and A-9). (**B**) Granulocytes stained with DAPI at 48 h post-injection. (B-1) Two nuclei were observed in the granulocyte cytoplasm. (B-2 and B-3) Occasionally, two or more nuclei, or deformed nuclei, were observed in the granulocyte cytoplasm (indicated by white arrows). Scale bar = 10 µm (A) or 20 µm (**B**).

**Figure 6:**
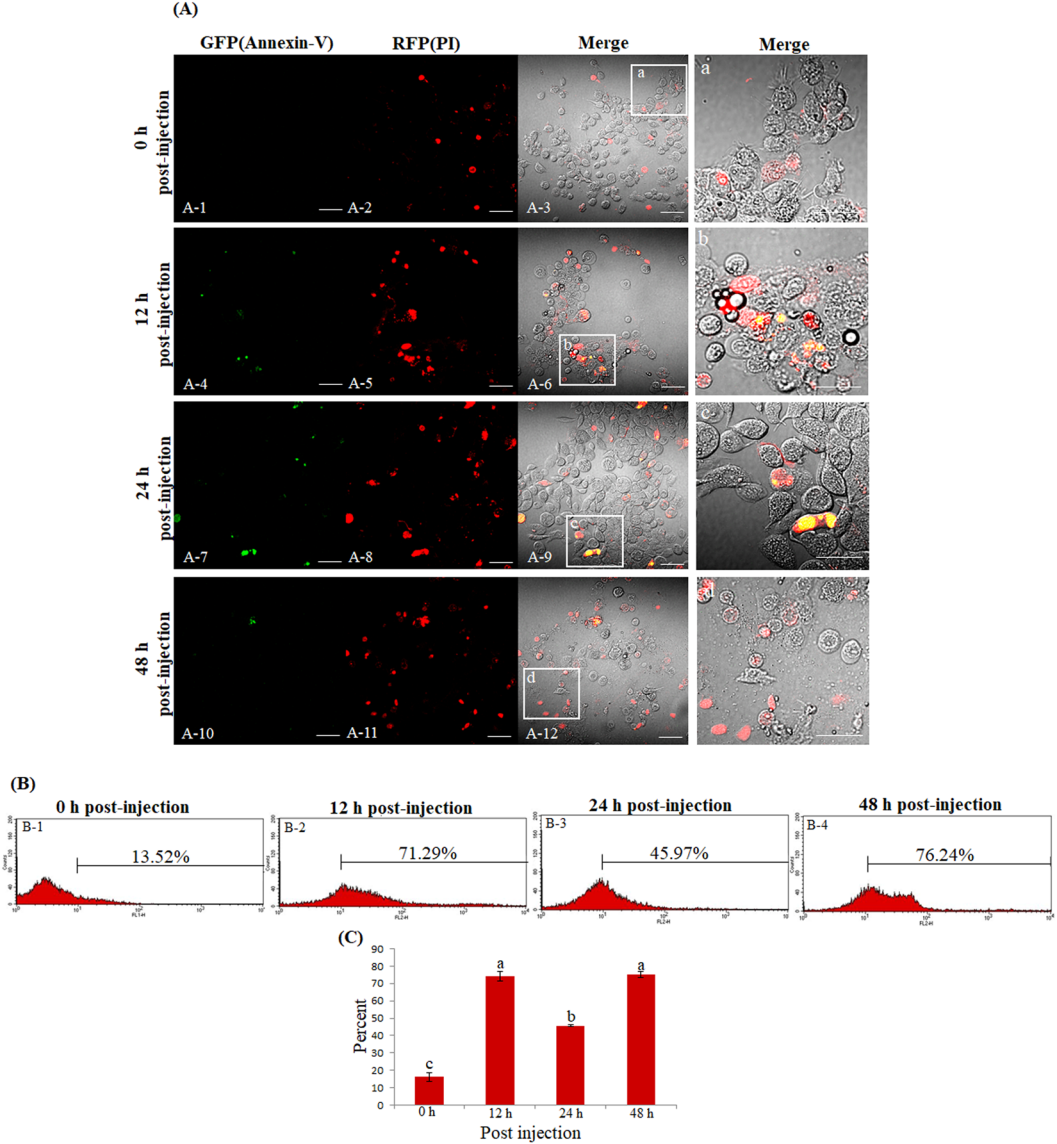
Microscopic and flow cytometric analysis of hemocytes stained with annexin V/Propidium Iodide (PI). (A-1, A-4, A-7, and A-10) The green fluorescent signal (annexin V) increased slightly. (A-2, A-5, A-8, and A-11) The red fluorescent signal (PI) increased significantly. Merged images of the red fluorescent signal (PI) and the DIC images are shown (A-3, A-6, A-9, and A-12). (a~d) The insets in panels A-3, A-6, A-9, and A-12 (indicated by white boxes) at a higher magnification. (**B**) Flow cytometric analysis at 0 h~48 h post-injection. (B-1 and B-2) At 12 h post-injection, 71.29% of granulocytes were stained with PI, compared with 13.52% at 0 h post-injection. At 24 h post-injection, the PI staining had decreased slightly, to 45.97%, but it increased again, to 76.24%, at 48 h post-injection. (**C**) The flow cytometry analyses were repeated three times. Scale bar = 50 µm (**A**) or 25 µm (insets).

Figure 2A shows hemocytes cultured with *E*. *coli* (see also Supplementary Movie 1). Some hemocytes were observed to be more aggregated and moving. As the incubation time increased, nets (amoeba-like hairs or extracellular traps) were produced by specific hemocytes, and various hemocytes were gathered together by these nets to form large clusters (Fig. 2A-1~A-6; amoeba-like hairs or extracellular traps indicated by black arrows). As shown in Fig. 2A-1, three groups of hemocytes (indicated by black circles) were ultimately drawn into one cluster by the nets (Fig. 2A-5 and A-6).

However, it was not possible to determine whether the gathering of the hemocytes described above was triggered by *E*. *coli*. To address this question, the hemocytes were activated with Sephadex beads (120 μm diameter), which can be easily observed with a DIC microscope (Fig. 2B, Supplementary Movie 2). As shown in Fig. 2A, the Sephadex beads were captured by the nets generated by specific hemocytes (Fig. 2B; nets indicated by black arrows in the yellow boxes). The Sephadex beads around the hemocytes were randomly labeled SP1, SP2, SP3, SP4, and SP5. As can be seen by comparing Fig. 2B-2 and B-3, SP1, SP2, and SP3 were drawn together and into the area indicated by the yellow box. In addition, SP1 eventually became completely surrounded by various hemocytes (Fig. 2B-9). The bead labeled SP4 also became incorporated into the cluster with SP1, SP2, and SP3 (Fig. 2B-4~B-9; indicated by the yellow box). Over time, all the Sephadex beads (SP1, SP2, SP3, and SP4) became surrounded by many hemocytes. By contrast, individual hemocytes were observed to be in contact with SP5, but it did not get drawn into the area indicated by the yellow box even though it was in a similar position as SP1. Therefore, nodulation by hemocytes seemed to occur randomly.

### Granulocyte activation by carboxylate-modified polystyrene latex beads

Next, we investigated which hemocytes produced the nets and participated in the various steps of the immune response, including encapsulation and nodulation. For this purpose, carboxylate-modified polystyrene latex beads, which can be easily observed with a DIC microscope, were used in place of pathogens, and real-time images of the hemocytes were collected. Figure 3A shows hemocytes stimulated with carboxylate-modified polystyrene latex beads for 12 h (Supplementary Movie 3). The nets produced by the hemocytes (indicated by black arrows) actively moved around the latex beads (indicated by red arrows) (Fig. 3A-1). Over time, the beads were engulfed by the nets and eventually could be seen within the cytoplasm of the hemocytes (Fig. 3A-2~A-4). However, not every carboxylate-modified polystyrene latex bead that encountered a net was phagocytosed. Thus, the movement of the activated hemocytes did not seem to correspond precisely with the movement of the beads.

Detailed observations were made using a high-magnification confocal microscope (Fig. 3B). At 4 h post-infection, the carboxylate-modified polystyrene latex beads could be observed within certain hemocytes (Fig. 3B-1; beads indicated by red arrows, and specific hemocytes indicated by white circles). We next observed which specific cells engulfed the latex beads in more detail (Fig. 3B-2~B-6). Figure 3B-2 shows resting granulocytes, which were usually round or oval in shape, with many polymorphic dark granules within the cytoplasm. At 2 h after stimulation with carboxylate-modified polystyrene latex beads, the granulocytes began to show morphological changes, including fan-like or amoeba-like protrusions of the plasma membrane (Fig. 3B-3; indicated by white arrows). Carboxylate-modified polystyrene latex beads were observed within the granulocyte cytoplasm at 4 h post-stimulation, and a large number of latex beads had accumulated in the cytoplasm of many granulocytes by 12 h post-stimulation (Fig. 3B-4 and B-5; beads indicated by red arrows, and nets indicated by white arrows). In addition, the nets were observed to become larger and wider over time (Fig. 3B-4 and B-5). At 48 h post-stimulation, many latex beads had accumulated in the cytoplasm of the granulocytes, but the nets could no longer be seen, and many granulocytes seemed to be inert (Fig. 3B-6; beads indicated by red arrows).

As shown in Fig. 2, the granulocytes appeared to attach not only to other granulocytes, but also to different types of hemocytes using these nets. We did not observe phagocytosis in any other cell type except for a small number of plasmatocytes (data not shown). In addition, we did not observe any immunological activity or morphological changes in any cell type other than granulocytes and a small number of plasmatocytes.

### Granulocyte lysosomes were activated by injection of *E*. *coli* particles

To investigate whether the vacuoles observed within the granulocytes were pathogen-related phagosomes, crickets were injected with *E*. *coli* particles, which are mainly used as markers of phagocytosis and fluoresce green when they reach acidified organelles such as intracellular lysosomes. At the same time, total hemocytes were stained with LysoTracker Red, which labels lysosomes. As shown in Fig. 4A-1, a green fluorescent signal (phagocytosed *E*. *coli* particles) was observed in the granulocyte cytoplasm immediately after injection of the particles. At the same time, a red fluorescent signal, which indicates activated lysosome formation, was also observed (Fig. 4A-2). At 4 h post-injection, highly polymorphic vacuoles could be seen in many granulocytes (Fig. 4A-4 and A-5). Merged images of the green fluorescent signal (phagocytosed *E*. *coli* particles) and the red fluorescent signal (activated lysosomes) are shown (Fig. 4A-6). At 12 h post-injection, the green fluorescent signal began to dim, while the red fluorescent signal remained (Fig. 4A-7~A-9). At 24 h post-injection, both fluorescent signals had dimmed (Fig. 4A-10~A-12). However, the red fluorescent signal in granulocytes was observed again at 48 h post-injection (Fig. 4A-13~A-15). Figure 4Aa~Ao shows the insets in panels A-1~A-15 (indicated by white boxes) at a higher magnification. Crickets that were injected with PBS buffer only were negative for red and green fluorescence at all time-points post-injection (Fig. 4B).

To quantify the green- and red-fluorescent signals in PBS- or *E*. *coli*-challenged crickets, hemocytes were analyzed by flow cytometry at 0~48 h post-injection (Fig. 4C). At 0 h post-injection, 2.08% of hemocytes were positive for green fluorescence, and this increased to 24.36% at 4 h post-injection (Fig. 4C-1 and C-2). The green fluorescent signal gradually decreased to 10.87% of hemocytes at 12 h, 3.98% at 24 h, and 1.74% at 48 h (Fig. 4C-3~C-5). These results indicate that *E*. *coli* particles were actively engulfed and digested by granulocytes and were completely cleared by 48 h post-injection. At 12 h post-injection, 69.54% were positive for red fluorescence, and the signal decreased to 5.78% of hemocytes at 24 h post-injection (Fig. 4C-1-1~C-4-1). Consistent with our microscopic observations, the red fluorescent signal increased to 30.25% of hemocytes at 48 h post-injection. By contrast, crickets that were injected with PBS were negative for green and red fluorescence at all time-points (Fig. 4C-1-2~C-5-2). The flow cytometry analysis was repeated three times (Fig. 4D).

### Reactivation of granulocyte lysosomes at 48 h post-infection

The reactivation of granulocyte lysosomes at 48 h post-infection was further investigated by microscopic observation (Fig. 5A and B). As shown in Fig. 4A, the green fluorescent signal (phagocytosed *E*. *coli* particles) and the red fluorescent signal (activated lysosomes) were seen at 4 h post-injection (Fig. 5A-1~A-3). The fluorescent signals had dimmed by 24 h post-infection (Fig. 5A-4~A-6). At 24 h post-infection, we observed that cells could be seen within the cytoplasm of the granulocytes (Fig. 5A-4~A-6; indicated by white arrows). This phenomenon was more apparent at 48 h post-infection (Fig. 5A-7~A-9; indicated by white arrows). In addition, the lysosomes around these engulfed cells at 48 h post-infection were activated (Fig. 5A-8). As shown in Fig. 4A-14, 4C-5-1, these microscopic observations seem to explain the increase in the red fluorescent signal at 48 h post-infection. To confirm this, the nuclei of the granulocytes were stained with 4′,6-diamidino-2-phenylindole (DAPI) at 48 h post-infection. As shown in Fig. 5B-1, granulocytes with two nuclei were frequently observed. Occasionally, granulocytes containing more than two nuclei, or deformed nuclei, were also observed (Fig. 5B-2 and B-3; indicated by white arrows).

### Necrosis-related granulocyte cell death at 48 h post-infection

As shown in Figs. 3B-6 and 5A-8, the accumulation of phagosomes in granulocytes altered their shape and induced cell death. At 48 h post-infection, the hemocytes were stained with fluorescein isothiocyanate (FITC)-conjugated annexin V and propidium iodide (PI) to confirm apoptotic or necrotic cell death (Fig. 6A). The green fluorescent signal (annexin V) only slightly increased between 12 h and 48 h (Fig. A-10, A-7, and A-10), but the red fluorescent signal (PI) showed strong staining at 12 h post-infection (Fig. 6A-5). At 24 and 48 h post-infection, the red fluorescent signal persisted (Fig. 6A-8 and A-11). Merged images of the red fluorescent signal (PI) and the DIC images are shown (Fig. 6A-3, A-6, A-9, and A-12). Figure 6Aa~Ad shows the insets in panels A-3, A-6, A-9, and A-12 indicated by the boxes at a higher magnification. These results indicate that the accumulation of phagosomes in granulocytes did not induce typical apoptotic cell death but necrosis-related cell death. To quantify the PI staining, hemocytes were stained and examined by flow cytometry at 0 h, 12 h, 24 h, and 48 h post-infection. At 12 h post-infection, 71.29% of granulocytes were stained with PI, compared with 13.52% at 0 h (Fig. 6B-1 and B-2). At 24 h post-infection, the PI staining had decreased slightly, to 45.97%, but again increased to 76.24% at 48 h (Fig. 6B-3 and B-4). The flow cytometry analysis was repeated three times (Fig. 6C).

## Discussion

The insect hemocyte types are classified according to their relative size, their morphology, and their uptake of various dyes. Using these methods, over the years, we have categorized the hemocytes of a variety of insects, while simultaneously improving techniques to identify and characterize insect immune cells^5,8,9,29,30^. However, insect hemocyte types are often misclassified depending on the conditions under which experiments are performed. For example, when hemocytes are dehydrated, fixed, and stained, they can be easily deformed by weak pressure, which changes their morphology. Occasionally, the nucleus of the hemocyte will be expelled, which can interfere with the classification of other hemocytes. Therefore, to accurately classify insect hemocytes *ex vivo*, techniques must be employed that maintain them *in vivo* state. For this purpose, we recently developed a method that allows insect hemocytes to be cultured *in vitro* for more than 3 weeks^30,31^. This allows us to more easily classify insect hemocytes *in vitro* and also to observe various immune responses in real time. Using these methods, we classified the circulating hemocytes of the cricket *Gryllus bimaculatus* based on their size, morphology, and function, into six types, granulocytes, plasmatocytes, prohemocytes, spherulocytes, coagulocytes, and oenocytoids. In addition, we show that granulocytes are the professional phagocytes in this insect and are a key player in the fight against pathogens.

Invading pathogens and parasites in insects are engulfed and killed by immune hemocytes^15–18^. Cellular defense mechanisms such as nodulation and encapsulation have been reported in insects, but these observations were reported *in vivo* and thus may not be accurate due to technical limitations. Using the *in vitro* culture technique for insect hemocytes that we developed, we could accurately observe cellular defense mechanisms such as nodulation, encapsulation, and phagocytosis of pathogens by cricket hemocytes. Our observations suggest that the cellular defense mechanisms of the cricket include the formation of sticky nets (amoeba-like hairs, pseudopods, and extracellular traps) by specific hemocytes (granulocytes) (Fig. 2A and B). The formation of these nets has also been reported in mammalian neutrophils^19,32–34^. Human neutrophils are a type of granulocyte. They are the most numerous type of immune cell and are one of the cell types known to phagocytose pathogens^35^. Recently, the sticky nets released by neutrophils to trap pathogens were reported to be composed of DNA and proteins released from the nucleus. The neutrophils that release these traps become aggregated with the pathogens and eventually die via a distinct mechanism of cell death known as NETosis^36–38^. In this study, we also observed many granulocytes dying after net formation, but we could not determine whether the mechanism of cell death involved DNA rupture, as is seen with human neutrophils, since NETosis cannot be directly observed in hemocytes cultured *in vitro*. The molecular mechanisms of NETosis by granulocytes in this insect therefore remain to be also elucidated. Although we could not confirm that the granulocyte death occurred by NETosis, it was at least associated with a necrotic mechanism, as shown in Figs. 3B-6 and 6. During real-time observation, granulocytes were observed to engulf latex beads and pathogens (Fig. 3B-6). This phenomenon was also observed in beetle immune cells, which could not withstand the pathogen load and eventually died^8^. In *Drosophila*, three types of hemocytes (crystal cells, plasmatocytes, and lamellocytes) have been reported. Plasmatocytes are professional phagocytes most similar to the mammalian macrophage, crystal cells secrete components necessary for the melanization of invading organisms, and lamellocytes are rarely seen in healthy larvae and are involved in the encapsulation of invading pathogens^39–41^. As shown in Figs. 2 and 3, the encapsulation of pathogens in the cricket also seems to depend on net formation by granulocytes. Although only granulocytes were observed to form nets, encapsulation of pathogens might involve all six types of hemocytes working together. To further investigate the immunological function of the other types of hemocytes, we need to develop techniques to isolate or cultivate these cells separately, but this will be challenging. As shown in Fig. 3A, phagocytosis was also mainly carried out by granulocytes. Phagocytosis begins when immune cells recognize various external signals expressed by the pathogens. These signals activate the immune cells, which then use nets to search for and trap the pathogens^42–44^.

As shown in Fig. 4, engulfment of pathogens by granulocytes led to activation of the lysosomes in their cytoplasm. Activation of granulocyte lysosomes started at 4 h post-infection and peaked at 12 h post-infection. This cycle of lysosome activation in immune cells has similarly been shown in other insects^8,9^. Thus, the process of cellular immunity in crickets occurs as follows: first, the granulocyte recognizes signals generated by pathogens; second, sticky nets are generated from the granulocyte plasma membrane; third, the granulocyte traps pathogens and engulfs them via nodulation, encapsulation, and phagocytosis; and fourth, the pathogens are degraded within activated lysosomes.

As these experiments proceeded, we observed that a considerable number of granulocytes became deformed and non-viable over time. The accumulation of lysosomes in the granulocytes altered their shape and might have induced the observed cell death. After participating in an immune response, immune cells often undergo cell death through mechanisms such as NETosis, autophagocytosis, apoptosis, or necrosis. This phenomenon has been reported in human neutrophils and insect granulocytes^10,25,27^. As shown in Fig. 6B, the percentage of PI-positive granulocytes increased during the course of infection with no lag period, whereas annexin V staining was not significantly increased compared with that for granulocytes from PBS-injected insects. These results indicated that the vacuolated granulocytes were undergoing necrosis. In addition, as shown in Fig. 5A-8, some cells were visible within the cytoplasm of the granulocytes, and activated lysosomes could be seen surrounding these engulfed cells. This result probably reflects a phenomenon in which necrotic granulocytes were removed by other healthy granulocytes. This process was not normal cell division (proliferation by autonomous mitosis), because the cells were strongly stained by the LysoTracker Red dye and two to three nuclei were observed within each cell. However, there are several reports in which the number of hemocytes increased by mitotic division after immune activation in several insects^8,45^. Thus, the mechanisms through which the granulocyte number is regulated *in vivo* need to be investigated.

## Materials and Methods

### Insect, hemocytes, and culture

The crickets, *Gryllus bimaculatus* were collected from humid dead trees near Kangwon National University. Approximately, 30 adults were maintained in a cage 80 × 60 × 90 cm at 25 ± 1 °C, 40 ± 10% relative humidity, and a long photoperiod of 16 h light: 8 h dark cycle under aseptic conditions using well-fermented sterile oak wood sawdust and paper boxes in a constant environment (CE) incubator (MIR-553; Sanyo Electric Biomedical, Japan). Through mass rearing, this species is recently being used as a food source for livestock such as chicken, pig, and cattle or insectivorous animals like reptiles and spiders. Hemolymph was directly collected from the hemocoel using a sterile glass Pasteur pipette (Haematokrit-kapillaren) by puncturing the cuticle dorsally and collecting the resulting material by capillary action. Then, around 500 μl of the hemolymph sample were placed into a sterile Eppendorf tube in the presence (v/v) of anti-coagulant solution (98 mM NaOH, 186 mM NaCl, 17 mM EDTA, and 41 mM citric acid, pH 4.5) and mixed well. The mixed samples were centrifuged at 1,000 g for 10 minutes. After centrifugation, the plasma was removed and then pellet was washed with sterile water used for *in vitro* experiments and hemocyte slide preparation. The hemocytes were cultured and maintained as previously described^30^. Briefly, hemocyte cultures were prepared by mixing ~10 μl hemolymph and ~5 ml of cell culture medium (Schneider’s Insect cell medium: 2 mg/ml tryptose phosphate, 10% inactivated fetal bovine serum, 0.52 mg/ml glucose, 30 mg/ml antimicrobiotics mixture, pH 7.0 with antibiotics) in petri dishes (16-well plates) and cultured at 25 °C for 7 days. Bacteria (*E*. *coli* K12), non-florescence carboxylate-modified polystyrene latex beads (1 µm in diameter; aqueous suspension, 10% solids content, Sigma; diluted 1∶10 in 0.15 M sterile phosphate buffered saline (PBS), Sigma), or non-florescence sephadex beads (50~150 µm in diameter, Sigma) were cultured with hemocytes to investigate cellular immune responses *in vitro*.

### Injection of potentially hazardous substances, staining hemocytes, fluorescence-activated cell sorting (FACS) analysis

The cold-anesthetized adult crickets were dorsally inserted into the hemocoel with pHrodo™ Green *E*. *coli* Bio Particles (Thermo Fisher) by a finely pulled glass needle (Haematokrit-kapillaren). The pHrodo™ Green dye is non-fluorescent at neutral pH (cytoplasm), but turns bright green upon acidification (lysosomes). Hemocytes were simultaneously stained with the acidotropic dye LysoTracker Red (7.5 nM; Molecular Probes) for 30 min at room temperature, washed three times with PBS and mounted. For DAPI (4′-6-diamidino-2phenylindole; 5 µg/ml) staining, hemocytes were fixed with cooled paraformaldehyde (4%) in PBS pH 6.5 for 15 min and stained with DAPI. Relative percentage of phagocytosis were analyzed by flow cytometry and visualization of stained granulocyte were confirmed by fluorescence microscopes at 1, 4, 12, 24 and 48 h post injection, including experiments with control larva hemocytes. To detected cell death, the annexin-V/Propidium Iodide (PI) kit according to the manufacturer’s instructions (Roche Diagnostics) has been used in microscopic or flow cytometric analyses. The green fluorescent signal (annexin-V) provides for detecting cellular apoptosis, while the red fluorescent signal (PI) is used to detect necrotic cell death. Briefly, 500 μl hemolymph samples were withdraw from the dorsal hemocoel and collected in a sterile Eppendorf tube in the presence (v/v) of anti-coagulant solution. Then, the annexin-V/PI working solution (100 µM) was added in the tube, incubated for 30 min at room temperature. After washing in PBS, the hemolymph samples were centrifuged at 1,000 g for 10 min at 4 °C and re-suspended in PBS and analyzed by microscopes or flow cytometry. The BD™ FACSCanto flow cytometer (BD Bioscience; San Jose, CA) were used for analyzing hemocyte staining according to protocols developed for this application using FACSDiva software from BD Biosciences. The green and red fluorescence were detected in the channel FL1 (530/30 band-pass) and the channel FL3 (610/20 band-pass), respectively. The percentages of green and red fluorescent were determined from 10,000 hemocytes every sample.

### Microscopy and statistical analysis

Cultured hemocytes were observed using a Leica microscope (Leica DM2500 upright and Leica DMI 3000B inverted fluorescence microscopes) and images including live hemocytes were acquired with a Leica photo camera (2048 × 1536 pixels resolution) using the LMD application software version 4.1. The Olympus FV1000 confocal microscope were used to take the confocal images analyzed with Olympus confocal software 2.0. For statistical tests, we used values by Student’s two-tailed t-test or one-way ANOVA at a probability (*p*) value of less than 5%.

## Supplementary information

Movie 1

Movie 2

Movie 3

Mvie C

## References

[1] Janeway CA, Medzhitov R (2002). Innate immune recognition. Annu. Rev. Immunol..

[2] Hoffmann JA (2003). The immune response of Drosophila. Nature.

[3] Tsakas S, Marmaras V (2010). Insect immunity and its signalling: an overview. Invertebrate Survival Journal.

[4] Bang K, Park S, Yoo JY, Cho S (2012). Characterization and expression of attacin, an antibacterial protein-encoding gene, from the beet armyworm, Spodoptera exigua (Hübner)(Insecta: Lepidoptera: Noctuidae). Mol. Biol. Rep..

[5] Lee M, Bang K, Kwon H, Cho S (2013). Enhanced antibacterial activity of an attacin-coleoptericin hybrid protein fused with a helical linker. Mol. Biol. Rep..

[6] JONES JACK COLVARD (1962). CURRENT CONCEPTS CONCERNING INSECT HEMOCYTES. American Zoologist.

[7] Gupta A (1985). Cellular elements in the hemolymph. Comprehensive insect physiology, biochemistry and pharmacology.

[8] Kwon H, Bang K, Cho S (2014). Characterization of the hemocytes in Larvae of Protaetia brevitarsis seulensis: involvement of granulocyte-mediated phagocytosis. PLoS One.

[9] Hwang S, Bang K, Lee J, Cho S (2015). Circulating hemocytes from larvae of the Japanese rhinoceros beetle Allomyrina dichotoma (Linnaeus)(Coleoptera: Scarabaeidae) and the cellular immune response to microorganisms. PloS one.

[10] Lee J, Hwang S, Cho S (2016). Immune tolerance to an intestine-adapted bacteria, Chryseobacterium sp., injected into the hemocoel of Protaetia brevitarsis seulensis. Scientific reports.

[11] Steiner H, Hultmark D, Engström Å, Bennich H, Boman H (1981). Sequence and specificity of two antibacterial proteins involved in insect immunity. Nature.

[12] Yakovlev AY (2017). Fat body and hemocyte contribution to the antimicrobial peptide synthesis in Calliphora vicina R.-D.(Diptera: Calliphoridae) larvae. In Vitro Cellular & Developmental Biology-Animal.

[13] Chaplin DD (2010). Overview of the immune response. J. Allergy Clin. Immunol..

[14] Brodin P, Davis MM (2017). Human immune system variation. Nature reviews immunology.

[15] Lavine M, Strand M (2002). Insect hemocytes and their role in immunity. Insect Biochem. Mol. Biol..

[16] Marmaras VJ, Lampropoulou M (2009). Regulators and signalling in insect haemocyte immunity. Cell. Signal..

[17] Dubovskiy I, Kryukova N, Glupov V, Ratcliffe N (2016). Encapsulation and nodulation in insects. Invertebrate Survival Journal.

[18] Vigneron, A., Jehan, C., Rigaud, T. & Moret, Y. Immune defenses of a beneficial pest: the mealworm beetle, Tenebrio molitor. *Frontiers in physiology* **10** (2019). 10.3389/fphys.2019.00138 PMC6422893 30914960

[19] Brinkmann V, Zychlinsky A (2012). Neutrophil extracellular traps: is immunity the second function of chromatin?. J. Cell Biol..

[20] Brinkmann V (2004). Neutrophil extracellular traps kill bacteria. Science.

[21] Altincicek B, Stotzel S, Wygrecka M, Preissner KT, Vilcinskas A (2008). Host-derived extracellular nucleic acids enhance innate immune responses, induce coagulation, and prolong survival upon infection in insects. J. Immunol..

[22] Zysk G, Bejo L, Schneider‐Wald B, Nau R, Heinz H (2000). Induction of necrosis and apoptosis of neutrophil granulocytes by Streptococcus pneumoniae. Clinical & Experimental Immunology.

[23] Esmann L (2010). Phagocytosis of apoptotic cells by neutrophil granulocytes: diminished proinflammatory neutrophil functions in the presence of apoptotic cells. J. Immunol..

[24] Fox S, Leitch AE, Duffin R, Haslett C, Rossi AG (2010). Neutrophil apoptosis: relevance to the innate immune response and inflammatory disease. J. Innate Immun..

[25] Iba T, Hashiguchi N, Nagaoka I, Tabe Y, Murai M (2013). Neutrophil cell death in response to infection and its relation to coagulation. Journal of intensive care.

[26] McCracken JM, Allen LH (2014). Regulation of human neutrophil apoptosis and lifespan in health and disease. Journal of cell death.

[27] Kobayashi SD, Malachowa N, DeLeo FR (2018). Neutrophils and Bacterial Immune Evasion. J. Innate Immun..

[28] Papayannopoulos V (2018). Neutrophil extracellular traps in immunity and disease. Nature Reviews Immunology.

[29] Cho S (2016). Ultrastructure Characterization of Hemcytes in Larvae of Protaetia brevitarsis seulensis. Korean journal of applied entomology.

[30] Hong M, Hwang D, Cho S (2018). Hemocyte Morphology and Cellular Immune Response in Termite (Reticulitermes speratus). J. Insect Sci..

[31] Duressa TF, Huybrechts R (2016). Development of primary cell cultures using hemocytes and phagocytic tissue cells of Locusta migratoria: an application for locust immunity studies. In Vitro Cellular & Developmental Biology-Animal.

[32] Villanueva E (2011). Netting neutrophils induce endothelial damage, infiltrate tissues, and expose immunostimulatory molecules in systemic lupus erythematosus. The Journal of Immunology.

[33] Kaplan MJ, Radic M (2012). Neutrophil extracellular traps: double-edged swords of innate immunity. J. Immunol..

[34] Kruger P (2015). Neutrophils: between host defence, immune modulation, and tissue injury. PLoS pathogens.

[35] Segal AW (2005). How neutrophils kill microbes. Annu. Rev. Immunol..

[36] Yipp BG (2012). Infection-induced NETosis is a dynamic process involving neutrophil multitasking *in vivo*. Nat. Med..

[37] Branzk N (2014). Neutrophils sense microbe size and selectively release neutrophil extracellular traps in response to large pathogens. Nat. Immunol..

[38] de Bont CM, Boelens WC, Pruijn GJ (2019). NETosis, complement, and coagulation: a triangular relationship. Cellular & molecular immunology.

[39] Wood W, Faria C, Jacinto A (2006). Distinct mechanisms regulate hemocyte chemotaxis during development and wound healing in Drosophila melanogaster. J. Cell Biol..

[40] Williams MJ (2007). Drosophila hemopoiesis and cellular immunity. J. Immunol..

[41] Vlisidou I, Wood W (2015). Drosophila blood cells and their role in immune responses. The FEBS journal.

[42] Strand MR (2008). The insect cellular immune response. Insect science.

[43] Rosales C (2011). Phagocytosis, a cellular immune response in insects. Invertebrate Survival Journal.

[44] Flemming A (2017). Insect Immunity: Mechanism of adaptive immunity found in the fruitfly. Nature Reviews Immunology.

[45] King JG, Hillyer JF (2013). Spatial and temporal *in vivo* analysis of circulating and sessile immune cells in mosquitoes: hemocyte mitosis following infection. BMC biology.

